# Biomaterial strategies for alleviation of myocardial infarction

**DOI:** 10.1098/rsif.2011.0301

**Published:** 2011-04-13

**Authors:** Jayarama Reddy Venugopal, Molamma P. Prabhakaran, Shayanti Mukherjee, Rajeswari Ravichandran, Kai Dan, Seeram Ramakrishna

**Affiliations:** Healthcare and Energy Materials Laboratory, Nanoscience and Nanotechnology Initiative, Faculty of Engineering, National University of Singapore, Singapore

**Keywords:** biomaterials, hydrogels, injectables, cardiomyocytes, mesenchymal stem cells, myocardial infarction

## Abstract

World Health Organization estimated that heart failure initiated by coronary artery disease and myocardial infarction (MI) leads to 29 per cent of deaths worldwide. Heart failure is one of the leading causes of death in industrialized countries and is expected to become a global epidemic within the twenty-first century. MI, the main cause of heart failure, leads to a loss of cardiac tissue impairment of left ventricular function. The damaged left ventricle undergoes progressive ‘remodelling’ and chamber dilation, with myocyte slippage and fibroblast proliferation. Repair of diseased myocardium with *in vitro*-engineered cardiac muscle patch/injectable biopolymers with cells may become a viable option for heart failure patients. These events reflect an apparent lack of effective intrinsic mechanism for myocardial repair and regeneration. Motivated by the desire to develop minimally invasive procedures, the last 10 years observed growing efforts to develop injectable biomaterials with and without cells to treat cardiac failure. Biomaterials evaluated include alginate, fibrin, collagen, chitosan, self-assembling peptides, biopolymers and a range of synthetic hydrogels. The ultimate goal in therapeutic cardiac tissue engineering is to generate biocompatible, non-immunogenic heart muscle with morphological and functional properties similar to natural myocardium to repair MI. This review summarizes the properties of biomaterial substrates having sufficient mechanical stability, which stimulates the native collagen fibril structure for differentiating pluripotent stem cells and mesenchymal stem cells into cardiomyocytes for cardiac tissue engineering.

## 1. Introduction

Cardiac tissue engineering promises to revolutionize the treatment of patients with end-stage heart failure and provides new solutions to the serious problems of heart donor shortage. Heart disease is one of the most common causes of death in the world [1]. Coronary heart disease (CHD) and heart failure continue to be significant burdens to healthcare systems in the western world. In the United States alone, there are 7.9 million survivors of myocardial infarction (MI) and 16.8 million people living with CHD (http://www.americanheart.org). The single most common cause of left-sided cardiac failure is ischaemic heart disease (also called coronary artery disease) with an episode of acute MI. Left ventricular dilatation is a well-recognized precursor of ventricular dysfunction and congestive heart failure after MI. The damaged left ventricle undergoes progressive ‘remodelling’ and chamber dilation, with myocyte slippage and fibroblast proliferation. The impairment of heart wall muscle is permanent because, after a massive cell loss owing to infarction, the myocardial tissue lacks significant intrinsic regenerative capability to replace the lost cells. The enlargement in ventricular volume leads to progressive structural and functional changes in ventricles (called ventricular remodelling). Ventricular remodelling is compensatory at the initial stages, but adds further inefficiency to the mechanical pumping of ventricular muscle, predisposing towards the final stage of congestive heart failure (CHF) [2], a condition in which the heart cannot pump sufficient amount of blood to meet the metabolic requirements of the body.

Current therapeutic strategies to treat CHF are limited to surgical transplantation, coronary artery bypass grafting (CABG), ventricular remodelling (resection), dynamic latissimus dorsi (LD) cardiomyoplasty, cardiac bioassist mechanical support and pharmacological intervention. These treatment options have significantly improved the quality of patient care, there are limitations to each approach. Heart transplantation is traditionally performed to treat intractable severe heart failure secondary to dilated and hypertrophic cardiomyopathy, but its use is restricted by the shortage of donors. The use of pluripotent stem cells to regenerate damaged heart tissue is being advocated as the new treatment for heart failure secondary to heart disease or severe MI. Myocardial dysfunction resulting from atherosclerosis-related MI is a widespread and important cause of morbidity and mortality among adults. The adult mammalian heart has limited regenerative capacity and therefore any significant myocardial cell loss is mostly irreversible and may lead to progressive loss of ventricular function and heart failure development. Despite the improvements in several pharmacological and surgical therapeutic measures, the prognosis for heart failure patients remains poor. Cellular cardiomyoplasty seems to reduce the size and fibrosis of infarct scars, to limit adverse post-ischaemic remodelling and to improve diastolic function.

Restoration of cardiac function by replacing diseased myocardium with functional cardiomyocytes (CMs) is an intriguing strategy because it offers a potential cure. There is an increasing evidence of experimental approaches to restore/regenerate failing myocardium. Two of the promising pathways are direct implantation of primordial type of cells into the injured heart and the replacement of portions of heart muscle with tissue-engineered bioartificial grafts. Both the techniques display advantages and limitations. The development of a bioartificial myocardium is a new challenge; in this approach, tissue-engineered procedures are associated with cell therapy. Organ decellularization for bioscaffold fabrication is a new investigated concept. Nanomaterials are emerging as the main candidates in ensuring the achievement of a proper instructive cellular niche with good drug release/administration properties [3]. Angiogenic cytokines can be used to induce vascularization within a cardiac patch [4]. Steffens *et al*. [5] physically immobilized vascular endothelial growth factor (VEGF) onto heparinized collagen matrices. The VEGF released from the matrices led to improved endothelial cell proliferation and angiogenesis in the chorioallantoic membrane. Alginate and alginate sulphate scaffolds enhance cardiac patch vascularization and viability by incorporating a mixture of prosurvival and angiogenic factors (SDF, IGF-1 and VEGF) by affinity binding to the scaffold [6]. Collagen scaffolds with covalently immobilized VEGF improved tissue formation by promoting cell proliferation within the graft of both *in vitro* and *in vivo*, thus leading to increased blood vessel density and reduced construct thinning in a rat model of right ventricle (RV)-free wall repair [7]. The direct transfer of pluripotent stem cells into heart has been reported to improve cardiac function of the recipient animals after myocardial injury [8,9]. Limitations are arrhythmogenicity, immunogenicity and tumorigenicity of the injected cells, as well as their insufficient potential to survive, engraft and differentiate into the cardiac cell phenotype. The majority of previously developed bioartificial matrices lack a heart-like microstructure. They are devoid of a vascular tree or microvasculature and are destined to die after implantation into the ischaemic heart. The functional improvement of the heart after cell or tissue transfer is usually attributed to secondary angiogenesis because of the lack of a convincing explanation of the mechanism of engraftment and participation in contractile activity [10]. In the last few years, other strategies have been evolved, aiming at restoring diseased areas of the heart. These approaches include cellular transplantation, as well as *in vitro* engineering of bioartificial myocardial tissues for alleviation of MI.

## 2. Myocardial infarction

MI results in the obstruction of blood supply to the heart muscle that leads to substantial death of CMs in the infarct zone followed by a rigorous inflammatory response and removal of dead cells by marrow-derived macrophages or mesenchymal stem cells (MSCs). Standard treatment of this debilitating disease comprises pharmacological protection of the heart, either from a primary injury or from secondary damage, as well as cardiovascular interventions, including percutaneous transluminal coronary angioplasty (PTCA) or heart transplantation. CMs are the most physically energetic cells in the body, contracting more than three billion times in an average human lifespan and pumping over 7000 l of blood per day along 160 000 km of blood vessels [11]. The control of heart contractions is almost entirely self-contained and can be attributed to the group of specialized CMs (pacemakers), the fastest of which are located in the muscle driven by the waves of electrical excitation generated by pacing cells that spread rapidly along the membranes of adjoining CMs and trigger release of calcium, which in turn stimulate contraction of myofibrils. Electromechanical coupling of myocytes is crucial for their synchronous response to electrical pacing signals [12]. According to theoretical simulations, addition of a heart patch border zone could decrease heart wall stress. In these simulations, added materials are non-contractile and have stiffness up to 200 per cent of the average stiffness of passive myocardium. The reduction in wall stress was calculated to be proportional to the fractional volume added, with stiffer materials improving this attenuation better [13]. On the other hand, the stiffness of heart muscle is 10 kPa at the beginning of diastole and 200–500 kPa at the end of diastole [14,15]. Most studies support the conclusion that cell implantation can improve contractile function of the heart. Clinical studies are underway to investigate the safety and feasibility of cell implantation in patients [16]. An alternative approach to deliver isolated cells into the heart is to use a tissue engineering strategy, in which a synthetic biodegradable patch is populated *in vitro* with MSCs and implanted onto the infarcted regions of MI for cardiac tissue regeneration [17–20].

## 3. Biomaterial strategies for alleviation of myocardial infarction

### 3.1. Properties of biomaterials

Tissue engineering approaches are designed to repair lost or damaged tissue through the use of growth factors, cellular transplantation, injectable biopolymers and biomaterial scaffolds. There are currently three biomaterial approaches for the treatment of MI. The first involves polymeric left ventricular restraints in the prevention of heart failure. The second uses *in vitro*-engineered cardiac tissue, which is subsequently implanted *in vivo*. The final approach entails injecting cells and/or a scaffold into the myocardium to create *in situ*-engineered cardiac tissue. Tissue engineering provides a solution to the problem of congenital or acquired heart defects that can be used to replace or reconstruct defective heart parts such as valves or vessels. A fabricated tissue engineering scaffold should be (i) highly porous with large interconnected pores (to facilitate mass transport), (ii) hydrophilic (to enhance cell attachment), (iii) structurally stable (to withstand the shearing forces during bioreactor cultivation), (iv) degradable (to provide ultimate biocompatibility of the tissue graft), and (v) elastic (to enable transmission of contractile forces) [21]. The biomaterial is used to create an engineered myocardial patch that should be easy to harvest, proliferate, non-immunogenic, and has the ability to differentiate into mature, functional CMs. Scaffold structure determines the transport of nutrients, metabolites and regulatory molecules to and from the cells, whereas the scaffold chemistry has an important role in cell attachment and differentiation. Mechanical properties of the scaffold should ideally match those of the native tissue, providing mechanical integrity of the forming tissue and supporting an *in vivo*-like mechanotransduction between cells and their environment [11].

**Figure 1.**
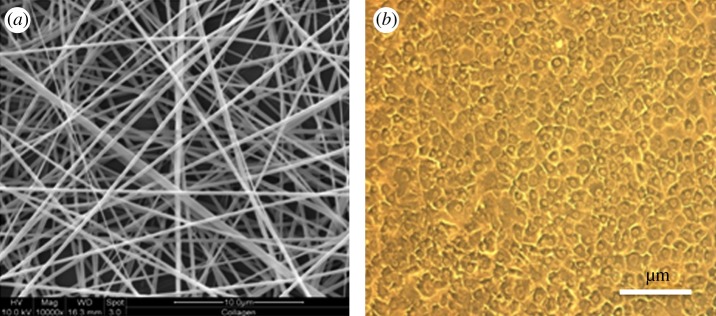
Nanofibrous scaffolds for cardiac tissue engineering. (*a*) Electrospun collagen nanofibres and (*b*) rat cardiomyocytes (CMs).

The ideal scaffold for implantation must meet several stringent criteria for tissue engineering. It must be biocompatible, reactive to non-foreign body, resistant to stress and strain, be sterilizable and match biomechanical characteristics of tissue it is replacing. Material degradation and resorption are other desirable properties, and the degradation products must be non-toxic and readily eliminate from the body. From a macroscopic perspective, the scaffold should be porous, with interconnecting pore structure to enable the accommodation of a large number of cells (CMs) and their organization into a functioning tissue (figure 1). Pore size of at least 50 µm is needed to allow the vascularization of scaffold after transplantation, to supply the seeded cells with nutrients and to remove secretions [22]. At the same time, the polymer scaffold should comprise good mechanical features to enable handling in cell culture during transplantation. Finally, the scaffold should be able to release growth factors, gene signals and other proteins, in a time-dependent manner. In general, biomaterial scaffolds for tissue engineering and regeneration can be divided into two categories: synthetic or biologically derived natural materials. Synthetic materials allow for precise control over properties such as molecular weight of the polymer, degradation time, mechanical properties and hydrophobic/hydrophilic properties. However, they may not interact favourably with cells as biologically derived materials do. The most popular synthetic materials are the degradable polyesters composed of lactide (PLA) and glycolide (PLG) and their copolymers (PLGA). Mukherjee *et al*. [23] studied the hydrophilic, biocompatible nanofibrous scaffolds made of poly(l-lactic acid)-co-poly(*ε*-caprolactone) (PLACL)/collagen that provides superior attachment and growth of adult cardiac cells favouring native myocardium-like alignment of newly seeded cardiac cells compared with purely synthetic PLACL scaffolds. Moreover, PLACL/collagen allows for cell–cell interaction without attenuating the functional activity of cells and cardiac-specific protein expression. These nanofibrous scaffolds have an elastic modulus of a magnitude nearing to that of native heart tissue in cardiac tissue engineering.

### 3.2. Extracellular matrix in myocardium

The myocardial collagen matrix mainly consists of type I and III collagens, which form a structural continuum. Collagen type I fibres mainly provide structural support and give the heart properties that include stiffness and resistance to deformation. The collagen type III fibres seem to play an important role as a link between contractile elements of adjacent myocytes, carrying some information useful for cell function. The lattice surrounding the myocytes comprises a complex network of structural proteins (collagen and elastic fibres) and adhesive proteins (fibronectin and laminin) within a hydrated proteoglycan and glycosaminoglycan-rich milieu [24,25]. Collagens such as types I, III, IV, V, VI and VIII have been identified in myocardium [26–28]. Cardiac fibroblast-synthesized type I and III collagens have different physical properties: type I collagen mainly provides rigidity, whereas type III collagen contributes elasticity [29]. The two type of collagens jointly support and tether myocytes in maintaining their alignment, tensile strength, shape and thickness in order to prevent rupture and contribute to the passive and active stiffness of the myocardium [30]. Changes in collagen type I : III ratio within the heart muscle may alter the tensile strength of the myocardium. Experimental observations have shown that, in the process of ischaemic heart disease, the myocardial extracellular matrix (ECM) is deeply altered, and the reserve of collagen type I, which is responsible for the structural support, can decrease from 80 to 40 per cent after MI [31]. A simultaneous increase in both myocardial collagen and diastolic chamber stiffness was attributed to increase the content of total collagen [32].

Natural polymers include both ECM proteins and derivatives (e.g. collagen) and materials derived from plants and seaweed (alginate). Natural polymers derived from ECM, such as Arg-Gly-Asp (RGD), collagen, gelatin on their surfaces can facilitate cell adhesion and maintain cell differentiation and advantageous for tissue engineering applications. However, these materials do not possess sufficient mechanical strength, unless they are chemically cross-linked to degrade rather rapidly in the body. In addition, batch-to-batch variations in material properties, as well as potential contamination when the materials are extracted from animal tissue, raise many concerns. Recombinant forms of human collagen and other materials are being produced, in order to avoid the use of animal products, by expressing them in cell lines including yeast [33]. Engineered heart constructs must develop systolic (contractive) force with appropriate compliance, and at the same time they must withstand diastolic (expansive) loads. Chachques *et al*. [22,34] performed clinical study in ischaemic patients, showed that bone marrow cell therapy associated with surgical implantation onto the epicardium of a cell-seeded collagen matrix type I (MAGNUM) prevented myocardial wall thinning, limited post-ischaemic remodelling and improved diastolic function. The use of MAGNUM seems to create a microatmosphere where exogenous and endogenous cells find the optimal microenvironment to repair with low scar formation. Cardiac tissue engineering (collagen matrix seeded with stem cells) emerges as a new therapeutic tool and extends even more amazing possibilities of cell therapies in cardiology, becoming a promising way for the creation of a ‘bioartifical myocardium’. Table 1 lists the mechanical properties (stiffness and tensile strength) of heart wall muscles and of the biomaterials investigated for myocardial tissue engineering [35–49].

**Table 1.** Potential biomaterials for cardiac tissue engineering. PGA, (glycolic acid); PLLA, poly(l-lactic acid); PHB, poly-(beta-hydroxybutyrate-co-betahydroxyvalerate); PPD, poly(para-dioxanone); TMC, 1,3-trimethylene carbonate; PDLLA, poly(d,l-lactide); POC, poly(1,8-octanediol- *co*-citric acid); PGS, poly(glycerol sebacate). n.a. not applicable.

polymer	elastomer (E)/thermoplastic (T)	Y modulus (stiffness)	tensile strength	degradation (month)	references
PGA	T	7–10 GPa	70 MPa	2–12	[35,36]
PLLA	T	1–4 GPa	30–80 MPa	2–12	[35]
PHB	E	2–3 GPa	36 MPa	degradable	[37]
PPD or PDS	E	0.6 GPa	12 MPa	6	[36,38]
TMC	E	6 MPa	12 MPa	degradable	[38]
TMC-PDLLA (50 : 50)	E	16 MPa	10 MPa	degradable	[38]
POC	T	1–16 MPa	6.7 MPa	degradable	[36,39]
PGS	E	0.04–1.2 MPa	0.2–0.5 MPa	degradable	[36,40,41]
collagen fibre (tendon–bone)	E	2–46 MPa	1–7 MPa	degradable	[42,43]
collagen gel (calf skin)	E	0.002–0.022 MPa	1–9 kPa	degradable	[44]
rat myocardium	E	0.001–0.14 MPa	30–70 kPa	n.a.	[45–47]
human myocardium	E	0.02–0.5 MPa	3–15 kPa	n.a.	[14,48,49]

### 3.3. Strategies of biomaterials

Poly(glycerol sebacate) (PGS) scaffolds were tailored to match the stiffness of heart muscle at the beginning of diastole (stiffness is 10–20 kPa) or the stiffness at the end of diastole (200–500 kPa) [50–52]. Once the engineered tissue construct is placed in the body, vascularization becomes a key issue for further remodelling in the *in vivo* environment. The pore size in the range of 50–100 µm was sufficient to allow vascularization of the scaffold following transplantation [53]. Radisic & Vunjak-Novakovic [54] suggested that larger (100–300 µm) pore size is necessary for vascularization and long-term survival of cardiac tissue constructs. The large pores could impair vascularization because endothelial cells are unable to bridge pores greater than a cell diameter [55,56]. A potential approach to address this problem is filling a highly porous scaffold with a cell-seeded and/or gene-containing collagen gels for vascularization [57,58]. Ott *et al*. [59] recently demonstrated that decellularized adult rat hearts retaining anisotropic structural and mechanical properties could provide a scaffold for cultured neonatal rat heart cells to regenerate nascent pump function of the engineered bioartificial heart. Engelmayr *et al*. [60] fabricated accordion-like honeycomb microstructure scaffold (PGS) to demonstrate the novel ability to yield tissue-engineered grafts with closely matched anisotropic mechanical properties compared with right ventricular myocardium of adult rats, while simultaneously promoting the preferential orientation of cultured neonatal rat heart cells in the absence of external stimuli.

Currently, the conduits or patches are made of Dacron polyester fabric, polytetrafluoroethylene (PTFE), glutaraldehyde-treated bovine pericardium or antibiotic preserved or cryopreserved homografts. Ozawa *et al*. [61] studied non-biodegradable PTFE and biodegradable non-woven PGA mesh, and biodegradable poly-l-lactide knitted or woven fabric with 50 per cent *ε*-caprolactone and 50 per cent l-lactide spongy polymer (PCLA) and transplanted the right ventricular outflow tract. The unique structure of PCLA patch with a spongy matrix favours *in vivo* cell colonization relative to other patches and also offers advantages relative to other biodegradable materials. Jin *et al*. [62] studied poly(lactide-co-*ε*-caprolactone) served as a mechanical ECM, where seeded bone marrow MSCs survived and differentiated into CMs, ultimately regenerating the myocardium and improving the cardiac function. Zmora *et al*. [63] developed three-dimensional porous scaffolds from alginate, using a simple, all-aqueous process based on freeze-drying techniques. The scaffolds were characterized by 90 per cent porosity and a pore size of 50–150 µm, depending on the freezing regimen. A more recent study shows the feasibility of bioengineering cardiac tissue within alginate scaffolds. After implantation onto rat-infarcted myocardium, the cardiac biografts stimulated intense neovascularization from the neighbouring coronaries and attenuated left ventricular dilatation and failure in an experimental model [50–52]. Several type of approaches for the transplantation of cell/biomaterials for MI and advantages/disadvantages are presented in tables 2 and 3 [64–82].

**Table 2.** Potential biomaterials and/or cell combinations used for cardiac tissue engineering. GAG, glycosoaminoglycan; PLLA, poly(l-lactic acid); PCL, polycaprolactone; PGA, poly(glycolic acid); POC, poly(1,8-octanediol-co-citric acid).

polymer	cells	approach	references
natural materials			
collagen	embryonic chick heart cells	epicardial heart patch	[64]
gelatin	cardiomyocytes	three-dimensional porous mesh	[65]
collagen-GAG	BM-MSCs	three-dimensional porous mesh	[66]
fibrin glue	no cells	ventricular heart patch	[67]
synthetic (degradable) materials			
PLLA	human ESC	three-dimensional porous mesh	[68]
PCL	cardiomyocytes	three-dimensional porous mesh	[69]
PGA	chondrocytes	three-dimensional porous mesh	[70]
polyurethane	mouse ESC	three-dimensional porous sponge	[71]
PGS		three-dimensional porous foam	[72]
non-biodegradable materials			
POC	HL-1 (cardiac cells)	scaffold application	[73]
PTFE	hUV ECs	cardiovascular graft	[74]
polypropylene	no cells	left ventricular constrain	[75]

**Table 3.** Approaches for using myocardial tissue engineering.

approach	advantages	disadvantages	reference
cellular cardiomyoplasty (injection of cells only, direct/indirect)	minimal invasive	lack of knowledge of cell function, cell loss, effect to only endocardium	[76,77]
*in situ* engineering (injection of cells and biomaterial)	biomaterial act as supporting matrix while cells will regenerate infarction	infancy stage	[78,79]
injection of biomaterials alone	matrix for homing autologous progenitor cells	immunogenicity, as only natural polymers have been suggested	[13]
left ventricular restraints (wrapping up the ventricle with biopolymer)	does not involve cell injection	prevents remodelling but does not repair damaged area	[80]
tissue engineering	ensures cells are delivered to desired area with minimal loss	involves open heart surgery, more work is required to determine suitable cell type and material	[81,82]

## 4. Potential cells for myocardial tissue engineering

### 4.1. Cell function in myocardium

Cell therapy is a novel treatment to prevent ventricular dilation and cardiac dysfunction inpatients suffered from MI. Cell-based regenerative therapy is undergoing experimental and clinical trials in cardiology, in order to limit the consequences of decreased contractile function and compliance of damaged ventricles following MI [3]. In cell-based therapy, isolated cell suspensions are directly injected into injured heart *via* pericardium, coronary arteries or endocardium. Direct injection of isolated cells avoids open heart surgery. However, it is difficult to control the location of grafted cells after transplantation. Cardiac myocytes are terminally differentiated cells with limited proliferative capacity and cannot compensate cell loss that occurs during MI or chronic heart failure. MI and heart failure resemble the most prevalent pathologies. In either case, the loss of CMs accounts for a decrease in myocardial function which can lead to total organ failure or trigger compensatory mechanisms such as hypertrophy of the remaining myocardium. Adult stem cells are rare and are technically difficult to isolate because of a lack of specific and accepted cell markers. Moreover, the process of differentiating some cell types, such as human embryonic stem cells, is difficult to control and carry the risk of teratoma. One exciting concept of a potential endogenous cell source in the cardiovascular system is of particular interest: the potential for ‘self-repair’ by induction of hyperplastic growth [83].

A crucial aspect of cardiac tissue engineering is its choice and the composition of cells in engineered heart constructs. Proposed cell sources for cardiac tissue engineering are provided in table 4 [84–97]. Clearly, cardiac myocytes have been the main cellular component for the heart. However, can the heart function without non-cardiac myocytes? Endothelial cells, fibroblasts, smooth muscle cells, neural cells and leucocytes comprise about 70 per cent of the total cell number in working myocardium [98] and undoubtedly play an important role in cardiac development and function [99]. Endothelial cells (ECs) and smooth muscle cells (SMCs), the main components of the vasculature, are not only necessary for transporting nutrition and oxygen, but also secrete growth factors and cytokines that are important for function of the heart. Cardiac myocytes stimulate endothelial cell production of platelet-derived growth factor-β (PDGF-β), which combines with PDGF-α to induce endothelial cell secretion of VEGF receptor Flk-1, which are critical components of angiogenesis. Troponin-T is important for effective CMs which contain contractile proteins as it regulates the force and velocity of myocardial contraction, and actinin is an important constituent of the contractile apparatus. Troponin-T is one of the essential proteins for contractile function and an indicator of differentiation in CMs. Nitric oxide secreted by endothelial cells causes vasodilatation of coronary vessels, exerts direct effects on myocardium and decreases isotonic twitch shortening isolated myocytes and enhances myocardial relaxation [100].

**Table 4.** Potential cells sources for myocardial tissue engineering.

source	reference
skeletal myoblasts	[84]
crude bone marrow	[85]
endothelial progenitor cells	[86]
haematopoietic stem cells	[87,88]
mesenchymal stem cells	[89]
smooth muscle cells	[90]
umbilical cord cells	[91]
fibroblasts	[92]
human embryonic stem cells	[93]
foetal cardiomyocytes	[65,83]
myocardial progenitors	[94–96]
cloned cells	[97]

### 4.2. Mesenchymal stem cells

Stem cells seem to be the only meaningful cell source to allocate enough myocytes for clinically relevant cardiac tissue engineering in the future. One gram of adult myocardium contains an estimated number of 20–40 million myocytes [101] and a typical MI that induces heart failure leads to a loss of approximately 50 g of the heart muscle [102]. In order to compensate such a loss, it seems likely that those engineered myocardium not only have a similar size but also contain equal amount of myocytes (50 g approx. 1–2 billion). CMs have the native contractile and electrophysiological properties of the heart muscle; they are difficult to obtain, expand and are allogenic cells. Disadvantages of embryonic stem cells include their potential for transformation into teratocarcinoma and other malignancies. In contrast, MSCs can easily be isolated from bone marrow, cultured, non-immunogenic and can readily be expanded in the laboratory, making them an attractive cell source for cardiac tissue engineering. MSCs have the greatest potential for use in cell-based therapy of human heart diseases, especially in MIs. The therapeutic potential of MSCs in myocardial repair is based on their ability to directly differentiate into cardiac tissues and on paracrine actions of factors released from them. However, the major obstacle in the clinical applications of MSC-based therapy is the poor viability of transplanted cells owing to harsh microenvironment-like ischaemia, inflammation and/or anoikis in the infarcted myocardium. Katritsis *et al*. [103] proved that intracoronary-treated MSCs reduced infarct size in human patients compared with controls. These results demonstrate the safety and feasibility of intracoronary MSC infusion in post-MI patients. Moreover, it seems that intra-myocardial delivery of MSCs during coronary bypass grafting and via catheter-based delivery system also is safe and feasible [104]. Therefore, MSCs may be used as a novel agent to induce regeneration and protection of infarcted myocardium.

Hare *et al.* [105] observed specific safety monitoring indicated that cell-treated patients have improved outcomes with regard to cardiac arrhythmias, pulmonary function, left ventricular function and symptomatic global assessment. These findings support the conduct of more extensive studies assessing the value of allogenic hMSCs for the treatment of cardiovascular disorders. Chen *et al*. [106] conducted a randomized study to investigate the effectiveness of intracoronary injection of MSCs in patients with acute MI. After occlusion of the infarct-related coronary artery, a suspension of autologous MSCs was directly injected into the target coronary artery through an inflated, over-the-wire balloon catheter. Cardiographic evaluation demonstrated significant variation in the group of patients who received MSCs in comparison with controls. The percentage of hypokinetic, akinetic and dyskinetic segments decreased in treated patients, while wall movement velocity over the infarcted region and left-ventricular ejection increased significantly in the MSC-treated group. Engrafted cells expressed the CM marker proteins, such as β-myosin heavy chain, α-actinin, cardiac troponin-T and phospholamban. Furthermore, engrafted cells develop into myofibres containing striated sarcomeric myosin heavy chain and cell-to-cell junctions. Cellular cardiomyoplasty using needle injections is emerging as a treatment option for individuals with chronic heart failure, but it may be limited by failure to regenerate cardiac mass in cardiac tissue engineering (CTE).

**Figure 2.**
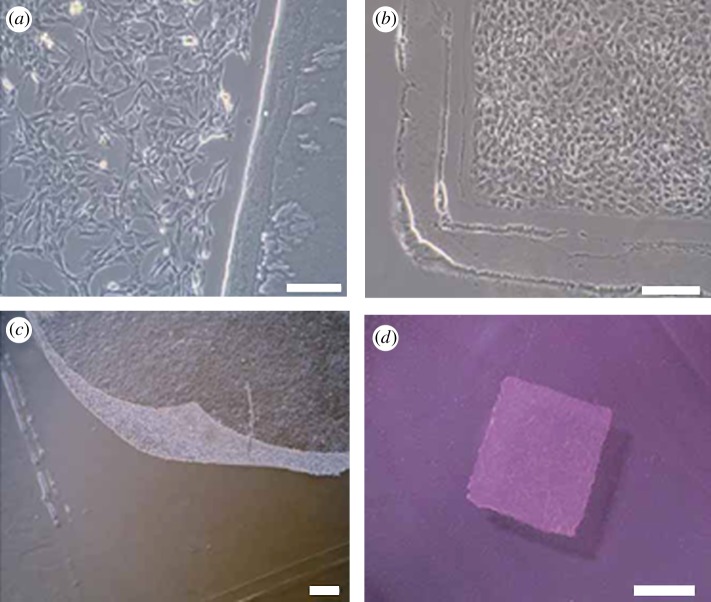
Preparation of monolayered mesenchymal stem cells (MSCs). (*a*) MSCs 2 days after seeding on a temperature-responsive dish, (*b*) cultured MSCs expanded to confluence within the square area of the dish by day 3, (*c*) the monolayered MSCs detached easily from the culture dish at 20°C and (*d*) the completely detached monolayered MSCs were identified as a 12 × 12 mm square sheet [107].

Miyahara *et al*. [107] developed cell sheets using temperature-responsive culture dishes to reverse cardiac wall thinning and prolong survival after MI, primarily owing to growth factor-mediated paracrine effects and by decreasing left ventricle wall stress after transplantation of cell sheets. These cell sheets allow cell–cell connections and maintain the presence of adhesion proteins because enzymatic digestion is not needed (figure 2). Placement of the adipose-derived MSC (ADMSC) sheets onto a scarred myocardium in rats resulted in diminished scarring and enhanced cardiac structure and function. Therefore, cell sheet transplantation may be a promising strategy for partial cardiac tissue reconstruction. In addition, owing to the increased secretion of angiogenic growth factors, VEGF, PDGF, basic Fibroblast growth factor (bFGF) and hepatocyte growth factor (HGF), the cardiac cell sheets containing ECs appeared to possess a significant innate potential for neovascularization even before transplantation for MI. Chachques *et al*. [108] suggested that cell transplantation offers promises induce angiogenesis, and to restore myocardial viability and regional ventricular function, therefore limiting remodelling for patients who have had a non-massive MI and probably for patients presenting with non-ischaemic dilated cardiomyopathy.

### 4.3. Bone marrow-derived cells

**Figure 3.**
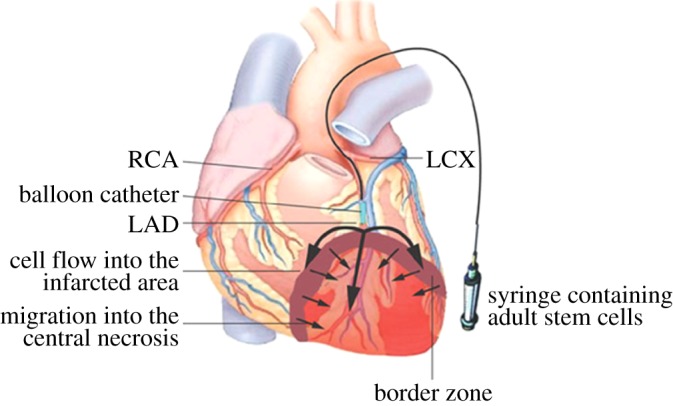
Transplantation of bone marrow cells into infarcted myocardium in humans. The balloon catheter enters the infarct-related artery and is placed above the border zone of the infarction. The catheter is then inflated and the cell suspension (including the patient's own cytokines) is infused at high pressure under stop-flow conditions. In this way, cells are transplanted into the infarcted zone through the infarct-related vessel system. Cells and cytokines infiltrate the infarcted zone. The arrows show the possible route of cell migration and cytokine infiltration. LAD, left anterior descending coronary artery; LCX, left circumflex artery; RCA, right coronary artery [112].

Experimental studies have shown that bone marrow-derived cells are capable of regenerating infarcted myocardium and inducing myogenesis and angiogenesis, which leads in turn to amelioration of cardiac function in mice and pigs [109,110]. Capsi & Gepstein [111] have given an excellently tabulated overview on the clinical trial results of using bone marrow stem cells in the treatment of acute and chronic heart diseases. Studies in animal models of ischaemia and phase I and II clinical trials suggested that delivery of haematopoietic stem cells (HSCs) and circulating endothelial progenitor cells, both originating from bone marrow stem cells, may result in the improvement of the ventricular function in ischaemic heart disease patients. Kocher *et al*. [109] demonstrated that an intravenous injection of human bone marrow donor cells into infarcted myocardium of rats resulted in a significant increase in neovascularization of post-infarction myocardial tissue, attenuation of CMs apoptosis and left ventricular remodelling. Strauer *et al*. [77] transplanted bone marrow cells (BMCs) directly into the infarcted zone of the myocardium. This was accomplished with the use of a balloon catheter, which was placed within the infarct-related artery. At this time, intracoronary cell transplantation via balloon catheter was performed, using six to seven fractional high-pressure infusions of 2–3 ml cell suspension, each of which contained 1.5–4 × 10^6^ mononuclear cells (figure 3). The results of the cell therapy group showed considerable improvement in left ventricular function. The transplantation of autologous BMCs as well as intracoronary approach represents a novel and effective procedure for the repair of infarcted myocardium [112].

### 4.4. Cardiomyocytes

**Figure 4.**
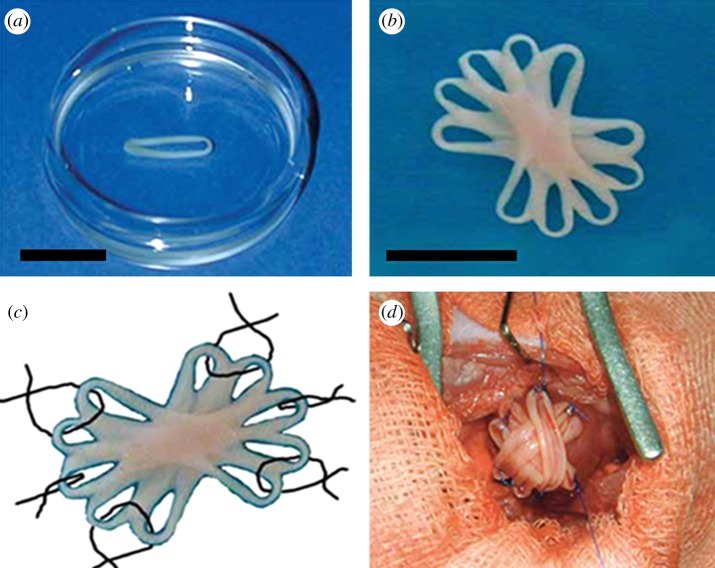
Construction of optimized engineered heart tissue (EHT). Stacking five single EHTs, (*a*) resulting in synchronously contracting multi-loop EHTs, (*b*) ready for *in vivo* engraftment, (*c*,*d*) six single-knot sutures served to fix multi-loop EHTs on the recipient's heart [57,58].

CMs have contractile and electrophysiological properties of the heart muscle, and they are difficult to obtain and expand for transplantation. CM sheets transplanted into ischaemic hearts were able to improve cardiac function and also bridged to form morphological communication through functional gap junctions within intact areas of the damaged myocardium [113]. Itabashi *et al*. [114] produced temperature-sensitive, resin-coated culture dishes to improve the clinical applicability of CM transplantation. The surface of these dishes became hydrophilic, when the temperature is lowered, and cultured CMs can be peeled off in sheets. Using this approach, a thin fibrin polymer membrane on the surface of culture dish is generated by reacting fixed concentrations of fibrinogen and thrombin. When CMs are cultured on these dishes, they secrete a variety of endogenous proteases that break down fibrin polymer membrane in approximately 3 days, making it possible to obtain CM sheets which are characterized by good cell viability and residence rates. Most studies report that less than 10 per cent CMs transplanted using a syringe resides stably in the heart, whereas few cells are lost after transplantation in which the CM sheets are transplanted subcutaneously. A further advantage of using CM sheets is that they can be layered to varying tissue thicknesses. These findings support that myocardial sheet formation is an important tool in future cell transplantation technology [115]. Zimmermann and co-workers [116,117] have developed a three-dimensional heart tissue model using a collagen matrix that allowed direct measurement of isometric contractile forces. Zimmermann *et al*. [57,58] developed a methodology to create engineered heart tissue (EHT) from neonatal rat heart cells. EHTs differ from classical scaffold-based tissue-engineered cardiac constructs in that they are originally made from heart cells, liquid collagen type I and Matrigel as well as growth supplements, reconstituted in circular moulds and subjected to mechanical strain (figure 4). Under these conditions, cardiac organoids develop spontaneously and show contractile as well as electrophysiological properties of working myocardium. The first EHT graft implantation experiments in healthy rats showed survival, strong vascularization and a sign of terminal differentiation to support contractile function of the infarcted heart.

### 4.5. Skeletal myoblasts

Cell transplantation for cardiac support and regeneration may repair the injured heart but is limited by poor effect in systolic function. This can be due to the lack of gap junctions between the native myocardium and the grafted cells. Myocardial injection of autologous myoblasts has been clinically performed and shown to produce some limited recovery from heart dysfunction. In these therapies using direct delivery of isolated cells, each cell differentiates and remodels in response to its surrounding environment, leading to tissue regeneration and functional repair. Today, the most widely used cell types for cardiac cell therapy in human patients are skeletal muscle-derived progenitors or myoblasts, and crude bone marrow mononuclear cells [118]. These cell types share advantages over other cells proposed for cardiac repair in that they are readily available, autologous and could be expanded *in vitro*. Layered skeletal myoblast sheets also provide improved left ventricular contraction, reduced fibrosis and prevented remodelling through recruitment of HSCs and the release of various growth factors [119]. Skeletal myoblasts do not fully differentiate into CMs *in vivo* after intramyocardial transplantation, and contracting myotubules do not operate in synchrony with the surrounding myocardium [120]. This is due, to the least part, to a lack of connexin activity and electrical coupling with the surrounding myocardial cells. Animal experiments also showed that the electrical coupling of skeletal myoblasts to resident CMs is increased when the skeletal cells are induced to overexpress connexin 43, indicating that there might be ways to overcome the arrhythmogenic obstacles [121]. The association of electrostimulation with cellular cardiomyoplasty could be a way to transform passive cell therapy into ‘dynamic cellular support’. The principle of electrophysiologic conditioning of skeletal muscle fibres (developed for dynamic cardiomyoplasty procedure) can be applied in cellular cardiomyoplasty [122]. Electrostimulation of both ventricles following skeletal myoblast implantation seems to induce the contraction of the transplanted cells and a higher expression of slow myosin, which is better adapted for chronic ventricular assistance. Patients with heart failure presenting myocardial infarct scars and indication of cardiac resynchronization therapy might benefit from simultaneous cardiac pacing and cell therapy [123]. The occurrences of ventricular perforation during intramyocardial transcatheter injection of myoblasts into thinned myocardium can be 5–7 mm; therefore, experience with intracardiac injection is critical for reducing complications. Autologous myoblast transplantation has the capacity to replace lost myocardial contractile cells and reverse the ventricular dilation [124,125]. Dib *et al*. [126] investigated the feasibility and safety of injecting myoblasts into a chronic myocardial infarct that can be thin, difficult to penetrate and potentially easy to perforate. Myoblasts can be safely and feasibly administered in patients by transcatheter technique in the hands of a trained investigator. Larger, randomized, double-blind, placebo-controlled and multi-centre clinical trials are warranted to further test this therapeutic approach for myocardial tissue engineering (MTE).

### 4.6. Umbilical cord blood stem cells

Human umbilical cord blood-derived stem cells (HUCBCs) might solve the problem of impaired stem cell function and number of sick and aged population. A specific advantage of HUCBCs is the immature immunogenicity of the mononuclear fraction, which significantly reduces the risk of rejection by host [83]. HUCBCs contain relatively high numbers of CD133^+^ and CD34^+^ progenitor cells. These cells have homing, myogenic and angiogenic potential that are relevant for myocardial repair [127]. The therapeutic effect of HUCBCs has been demonstrated in animal models of hind limb ischaemia and stroke [128,129]. The use of HUCB stem cells to repair the infarcted myocardium might be of importance for elderly people in whom the availability of autologous stem cells is limited for cell therapy. Intramyocardial injection of HUCBCs preserves LV function following infarction. The use of a cell-seeded collagen matrix combined with cell injection prevents ventricular wall thinning and limits post-ischaemic remodelling. This tissue engineering approach seems to improve the efficiency of cellular cardiomyoplasty and could emerge as a new therapeutic tool for the prevention of adverse remodelling and progressive heart failure [130]. Improved methods for stem cell expansion, storage and induction of immune tolerance would increase the prospect of using HUCB cells to treat MI patients, especially those who need it urgently.

### 4.7. Adipose-derived stem cells

Fat is abundant in most individuals, allowing a simpler and more efficient harvesting, as adipose tissue has a higher stem cell yield than bone marrow [131], and diminishing the need of *in vitro* expansion. Adipose-derived stem cells (ADSCs) can easily be isolated and cultured *ex vivo* and express markers associated with mesenchymal and perivascular cells including STRO-1, CD146 and 3G5, maintaining their characteristic multipotency to differentiate into chondrocytes, osteoblasts, endothelial cells and CMs. The differentiation capacity and paracrine activity of these cells made them an optimal candidate for the treatment of a diverse range of diseases from immunological disorders as graft versus host disease to cardiovascular pathologies peripheral ischaemia [132]. Four different possible fates of ADSCs are described by Choi *et al*. [133] such as: (i) differentiating into cardiac muscles by direct contact with adjacent rCM; (ii) differentiating into SMCs that have migrated to and surrounded immature vessels; (iii) adipogenic differentiation; and (iv) secreting proangiogenic factors to recruit endogenous endothelial cells. In general, transplanted cells can act upon the damaged heart in several ways, such as increasing myocardial perfusion, enhancing endogenous cell survival, attracting progenitors and regulating tissue fibrosis. Rigol *et al.* [134], studied a pig model of ischaemia reperfusion, injected passage three ADSC either via a transendocardial catheter or through intracoronary infusion one week after infusion of MI. Transplanted cells engrafted, differentiated to smooth muscle cells and increased the density of arterioles to a similar degree by either approach, although they were not able to demonstrate significant cardiac function. Okura *et al*. [135] showed that the phenotype of hADMSCs could be changed to cardiac-like cells (CLCs) by the induction of dimethylsulphoxide. These hADMSCs-derived CLCs engrafted into a scarred myocardium and differentiated into CMs for cardiac tissue regeneration. Hypoxia-treated ADSC co-culture with early postnatal CMs (2–5 days) have been shown to enhance blood vessel growth not only by the production of paracrine factors, but also by promoting the differentiation of existing cardiac progenitor cells to endothelial cells [136,137]. The different cell sources, principally skeletal myoblasts, ADSCs and BMCs, should provide the angiogenic and ventricular remodelling in myocardial regeneration.

## 5. Biomaterial strategies for alleviation of myocardial infarction

Several groups have reported encouraging results with various techniques to construct beating cardiac patches for transplantation. However, assembling vascularized three-dimensional myocardial tissues remains an enormous challenge. Most studies support the notion that cell implantation in models of MI can improve contractile and mostly diastolic function. Presently, clinical studies are underway to investigate the feasibility of cell implantation in patients for MI. An alternative approach for the injection or infusion of isolated cells into heart is the design of artificial cardiac muscle constructs *in vitro* for later implantation *in vivo*. Several principally different cardiac tissue engineering approaches have been developed. These are: (i) seeding of CMs on preformed polymeric scaffolds, which may function as organ blueprints, (ii) stacking of CMs monolayers to form cardiac muscle-like tissue without additional matrix material, and (iii) entrapping of CMs in a cardiogenic environment to support self-assembly into functional myocardium. These tissue engineering concepts have been tested in animal models showing survival and growth of engrafted heart muscle surrogates [120].

**Figure 5.**
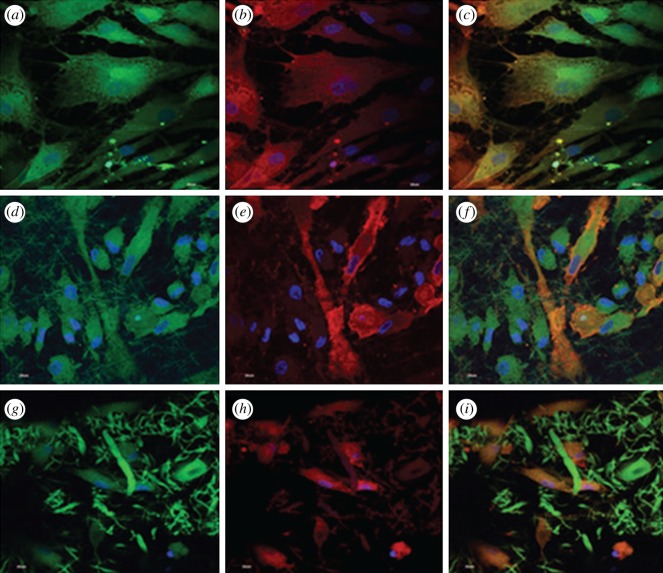
Core/shell (PGS/gelatin) fibrous structure for regeneration of myocardial infarction (MI). Dual immunocytochemical analysis for (*a,d,g*) the expression of MSC marker protein CD 105 and (*b,e,h*) cardiac marker protein actinin in the co-culture samples and (*c, f, i*) the merged image showing the dual expression of both CD 105 and actinin; on (*a,b,c*) the TCP, (*d,e,f*) gelatin nanofibres and (*g,h,i*) PGS/gelatin core/shell fibres at 60× magnification. Nucleus stained with DAPI [141].

Restoration of heart function by replacement of diseased myocardium with functional CMs is an intriguing strategy because it offers a potential cure [138]. Shimizu *et al*. [139] cultured CMs on temperature-responsive polymer poly(*N*-isopropylacrylamide) (PIPPAm) by electron beam exposure, producing surfaces that are slightly hydrophobic and cell-adhesive under culture condition at 37°C and change reversibly to hydrophilic and non-cell-adhesive below 32°C owing to rapid hydration and swelling of grafted PIPPAm. This unique surface change allows for cultured cells to detach spontaneously from these grafted surfaces simply by reducing culture temperature. The CM sheets, which readily detach from PIPPAm-grafted surfaces and transfer onto rigid culture surfaces or other CM sheets, stop their intrinsic beating temporarily but spontaneously recover within a few days. The cell sheet manipulation technology (cell sheet engineering) using temperature-responsive cell culture surfaces has been shown to be very useful for fabricating electrically communicative, pulsatile cardiac grafts both *in vitro* and *in vivo*. This technology should have enormous potential for constructing *in vitro* three-dimensional heart tissue models and for improving viable functional graft materials for clinical tissue repair. Synthetic polymers are essential materials for tissue engineering not only owing to their excellent processing characteristics, which can ensure off-the-shelf availability, but also advantages of biocompatible and biodegradable properties. These polymers have predictable and reproducible mechanical and physical properties (e.g. tensile strength, elastic modulus and degradation rate), and can be manufactured with great precision. Degradability is generally a desired characteristic in tissue engineering substrates because the second surgery to remove them (such as a heart patch) would be averted if the substrate could be removed by physiological system of the host body. The elastomer PGS, recently developed for soft tissue engineering, represents a feasible candidate that fulfils all of the above criteria for cardiac tissue engineering [140]. Elastomer-based grafts may facilitate compliance of matching, thereby ameliorating the lifespan of the patients. Scaffolds composed of PGS are elastic and reversibly deformable and are thereby conducive to contracting CMs and engineered myocardium [41]. Other desirable properties of PGS include control of its mechanical properties, the capacity to form a variety of geometries on the macro- and micro-scales, and low inflammatory response and fibrotic encapsulation, coupled with retention of mechanical strength during degradation *in vivo*. NUSNNI Laboratory fabricated PGS/gelatin core/shell fibres by coaxial electrospinning for cardiac tissue engineering. In PGS/gelatin core/shell fibres, PGS is used as a core polymer to impart the mechanical properties and gelatin as a shell material to achieve favourable cell adhesion and proliferation [141]. The expression of MSC-specific marker protein CD 105 by the MSCs cultured in the co-culture (MSC/CM) environment on TCP, gelatin and poly(glycerol sebacate)/gelatin core/shell fibres (figure 5*a,d,g*). Figure 5*b,e*,*h* shows the expression of cardiac marker protein actinin. MSCs differentiate into cardiogenic lineage to express both CD 105 and cardiac-specific marker protein actinin. Dual expression of CD 105 and actinin by MSCs after cardiogenic differentiation is observed in figure 5*c,f*,*i*. In PGS/gelatin core/shell fibres (figure 5*i*), more cells express actinin markers indicating that the differentiation is higher in these scaffolds than in gelatin nanofibres (figure 5*f*). The observed results proved that the PGS/gelatin core/shell fibres have potential biocompatibility and mechanical properties for fabricating nanofibrous cardiac patch and would be a prognosticating device for the restoration of myocardium.

Many studies have been published using different synthetic or naturally occurring biomaterials for the application in MTE. Among the natural polymers, collagen, alginate and gelatin have been under intensive investigation for MTE by research groups in Germany [107,110], and Israel [63,83,142], respectively. Among the synthetic polymers, PGA, and copolymers with poly(lactic acid) (PLA) and poly(*ε*-caprolactone) (PCL) have been studied systematically using bioreactors for MTE at MIT [143,144] and Harvard [145,146]. The thermo-responsive polymer PIPAAm was applied to cardiac tissue engineering by researchers in Japan [147]. Cardiac devices made from non-degradable polymers have been under intensive animal and human trials by surgeons mainly from the USA [148,149]. Seikiya *et al*. [150] attempted to control the vascularization processes *in vitro* to create thicker functional tissues. When ECs were co-cultured within cardiac cell sheets, angiogenesis-related gene expression and the formation of EC networks were observed *in vitro*. The EC networks were maintained within the cell sheets after harvest from temperature-responsive culture dishes and matured to form tubularized vascular networks after *in vivo* transplantation. The formation of myocardial tubes with potential circulatory support could be created with cell sheet engineering. These new myocardial structures present a possible core technology for the creation of engineered tissues capable of acting as independent cardiac-assisting devices for CTE. Narmoneva *et al*. [151] observed that the presence of EC networks profoundly improves CM survival and organization by maintaining a minimum intercapillary distance to provide oxygen and nutrients. Therefore, the presence of ECs may be directly correlated with CM function.

**Figure 6.**
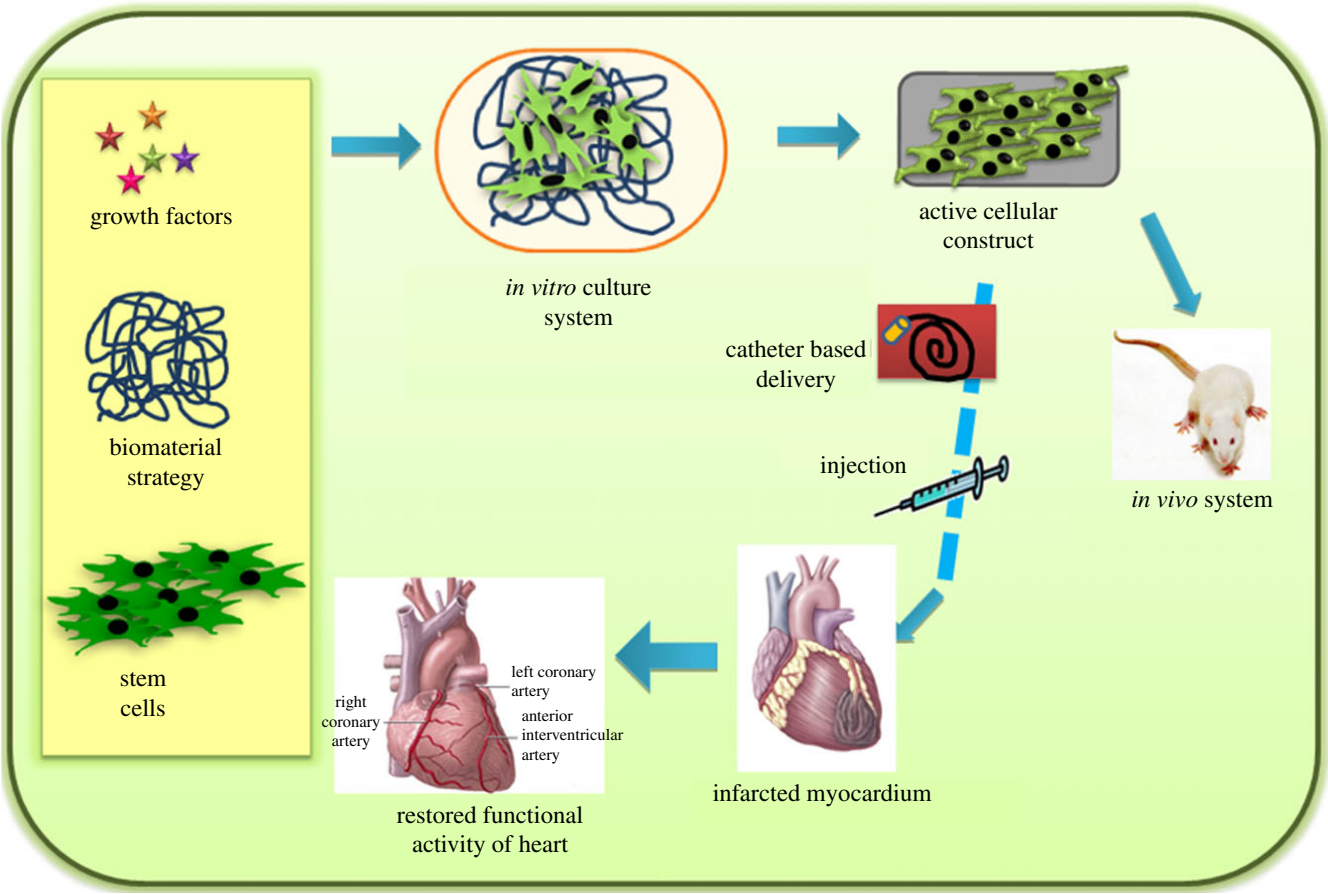
Schematic of the regeneration of MI.

Chen *et al*. [50–52] demonstrated that the porous tissue scaffold sandwiched with multi-layered sheets of MSCs serve as an effective cardiac patch to restore the dilated LV and improve heart functions in a syngeneic rat model with an experimentally chronic MI. Cells derived from rat ventricular muscle seeded into a biodegradable gelatin mesh (Gelfoam) can grow in three dimensions, proliferating to form cardiac-like tissue. Gelatin grafts persisted over a five week course after implantation either into the subcutaneous tissue or onto the myocardial scar of adult rats. These grafts maintained spontaneous and rhythmic contractility, but the effect of this graft on ventricular function after myocardial scarring remains uncertain. Matrigel constructs seeded in perfusion had physiologically high and spatially uniform cell density throughout the perfused construct volume, whereas constructs seeded in dishes had most cells located approximately 100 µm thick layers at the top surface. Cultured cells expressing cardiac-specific differentiation markers (sarcomeric α-actin, sarcomeric tropomyosin and cardiac troponin) were present throughout the perfused constructs and only within a approximately 100 µm thick surface layer in dish-grown construct [152]. Kofidis *et al*. [153] engineered a novel and promising type of myocardium-like tissue that resembles native cardiac muscle in many aspects. In addition, artificial myocardial tissues (AMTs) might serve as a basis for the development of tissue, which is capable of replacing human myocardium in many disease states of the failing heart. The figure 6 shows schematic diagram of the future progress in stem cell technology, as well as discovery of factors responsible for proliferation of MSCs and adult CMs, and when combined with suitable techniques of gene transfer might allow for the production of autologous artificial myocardium-like tissue/injectables capable of correcting infarcted myocardium and restoring impaired heart function. Finally, vascularization of *in vitro*-engineered tissues might result in the generation of a complete bioartificial heart.

### 5.1. Bioreactor system

A bioreactor provides a controllable biochemical and biophysical environment during the culture of engineering tissues. Compared with static culture, bioreactors enable control and monitoring of mass transport of oxygen, growth factors and nutrients, and biophysical stimuli such as cyclic stretch, hydrodynamic forces and electrical stimulations. These stimuli have shown to improve homogeneity of the construct, enhance the production of ECM components and to improve the functional properties of the construct [154]. A perfusion bioreactor provided pulsatile flow physiologically relevant shear stresses and the flow rate was constructed by the incorporation of a normally closed solenoid valve that was driven to open at a frequency of 1 Hz at the output from the perfusion chamber. Cultivation under pulsatile flow enhanced contractile properties of the cardiac constructs in bioreactor system [155]. Akins *et al*. [156] have shown three-dimensional contractile CM aggregates on polystyrene beads in a rotating bioreactor system. Papadaki *et al*. [157] engineered three-dimensional cardiac constructs for *in vitro* impulse propagation studies using biodegradable polymer (PGA) scaffolds in rotating bioreactor systems. Li *et al*. [158] have demonstrated that tissue-engineered cardiac graft transplantation using biodegradable gelatin mesh replaced both myocardial scar and right ventricular outflow track defects.

### 5.2. Injectable biomaterials

Recently, injectable tissue engineering scaffolds have been constantly pursued, aiming at minimally invasive surgery. Alginate, a negatively charged polysaccharide from seaweed that forms hydrogels in the presence of calcium ions, is being developed for tissue engineering in native and modified forms for cardiac tissue engineering. Compared with other materials, a major advantage of the injectable alginate biomaterial solution is its non-thrombogenicity. Tsur-Gang *et al*. [159] recently showed that a solution of calcium cross-linked alginate biomaterial with cell adhesion peptides, containing the sequences RGD and YIGSR, or a non-specific peptide (RGD), can be injected via a needle into the infarct, where it undergoes phase transition into hydrogel for left ventricular remodelling and function of post-MI. This alginate hydrogel implant provides temporary physical support to the damaged cardiac tissue by replacing some of the functions of damaged ECM while preventing adverse cardiac remodelling and dysfunction after recent and old MI in rat. With time, the dissolvable hydrogel gradually disappears, and water-soluble alginate chains are evacuated and excreted by the kidneys. The injectable biomaterial is delivered into the infarct zone. Christman *et al*. [78] first demonstrated improved cell survival when transplanted cells delivered in an injectable scaffold were compared with the typical cellular cardiomyoplasty technique. The injectable polymer fibrin glue was also shown to induce neovascularization within the ischaemic myocardium and to reduce infarct expansion. More interesting is the observation that injection of fibrin glue with or without skeletal myoblast preserved LV geometry and cardiac function in an acute MI model [79]. Anatomically, injectable gels have been applied as an endoventricular heart patch. It has been shown that injection of fibrin glue preserves left ventricular geometry and prevents a deterioration of cardiac function following MI [153]. The injectable gels lack sufficient stiffness for the application in human tissues. The stiffness of a variety of possible materials is in the range of 10 Pa to 20 kPa, such as fibrin (approx. 50 Pa), Matrigel (30–120 Pa), type I collagen gels (100 Pa to 6 kPa for 1–3 mg ml^−1^) [152,153], polyethylene glycol (1–3 kPa) [160] and alginate (100 Pa to 6 kPa) [159]. Injectable biomaterials can reduce wall stress by increasing the scar thickness and stabilizing the chamber size [161]. The injectable alginate increases scar thickness and provides physical support for improved healing and repair. The ability to deliver biomaterial into the infarct by intracoronary injection can revolutionize patient treatment after MI and could prevent mechanical complications, heart failure and death [162]. These materials are softer than human heart muscles at the end of diastole, the stiffness of which is approximately 50 kPa in normal hearts or 200–300 kPa in CHF hearts. Hence, it is unlikely that they could provide sufficient mechanical support to the diseased heart. In future, modifying the hydrogel with biopolymers and growth factors to increase the mechanical properties and having potential elastic properties suitable for cardiac tissue engineering could be possible.

### 5.3. Cardiac supporting devices

MI is caused by a significant reduction in coronary blood supply to an area of the heart over a sustained period, eventually forming non-contractile ability compared with the healthy heart. Representative cardiac restraint devices include Marlex mesh (polypropylene) [163], Merselene mesh (knitted polyester) [164], BioVAD [165] and MAGNUM [34]. A commercially available cardiac support device from Acorn Cardiovascular Inc. (knitted polyester) manufactured from a very common polymer PTFE has been used as a wrap around the cardiac ventricle. Several issues still need to be addressed for the success of MTE. First, electrical coupling between the cells is required, in order to ensure that cells on the graft or patch beat in synchrony. Second, electrical coupling between the construct and native myocardium for simultaneous beating is still of concern. It has been reported that cell sheet engineering has overcome this problem, where graft integration and no arrhythmias were reported [166–168]. MTE will hopefully lead to improvement in function of the diseased myocardium as it integrates with the heart, reducing the morbidity and mortality of patients with heart failure [169].

## 6. Conclusions

Cardiovascular tissue being a hierarchically organized tissue, the delivery of cytokines and bioactive proteins in a controlled and timely manner through nanostructured materials with suitable mechanical properties could be the ideal approach for improving the cardiac function. The three established mechanisms involved in myocardial repair are the CM regeneration, vasculogenesis and paracrine actions. Cardiac functional improvement can be accomplished using growth factors such as VEGF, which can mediate the angiogenic effect, and IGF-1, which can mediate the apoptotic effect. Even embryonic stem cells could be differentiated into CMs by cardiac paracrine pathways mediated through TGF-β and BMP-2, and patients benefit after its transplantation to the diseased heart. Nanoengineered platforms that combine both ‘smart’ biomaterials and stem cells can provide the necessary stimulatory effects for differentiation of stem cells into CMs. At the same time, the encouraging preliminary results of cardiac tissue engineering experiments in small animal models helped in widening new theories of myocardial tissue regeneration. Material development involving injectable polymeric hydrogels and matrices compatible for catheter delivery allows for the establishment of cellular environments more suitable for cardiac regeneration. For tissue engineering technology to be more effective in human patients, it is critical that we can create 1 cm^2^ muscular patch/device to repair infarct myocardium. CMs are very sensitive to prolonged ischaemia and may respond in necrosis and apoptosis of the engineered myocardial graft. In coming years, scientists and engineers will bring about new insights into this fascinating field and hopefully answer questions regarding the optimal scaffold, a cell source that is autologous and unlimited, optimized methods to generate large tissue constructs with relevant contractile properties and eventually surgical techniques to replace or substitute diseased myocardium with engineered cardiac muscle constructs. The rapid innovations in tissue engineering research and stem cell biology will accelerate and optimize engineered tissue assembly; they may bring us to the point of being able to create an alternative tissue/injectables to repair or replace damaged heart muscle for the alleviation of MI.
